# Boosting Computational Catalysis and Chemical Reactivity with Artificial Intelligence

**DOI:** 10.1021/jacs.5c17786

**Published:** 2026-02-20

**Authors:** Konstantinos D. Vogiatzis, Clémence Corminboeuf, Ainara Nova, Kjell Jorner, Johannes Kästner, Markus Meuwly, Philippe Schwaller, Victor Böttcher, Maria Drosou, Edvin Fako, Hannes Hoppe, Zarko Ivkovic, Nestor Iwanojko, Dimitrios A. Pantazis, Stefan P. Schmid, Kalman Szenes, Auguste Tetenoire, Markus Reiher

**Affiliations:** † Department of Chemistry, 4285 University of Tennessee, Knoxville, Tennessee 37996, United States; ‡ Institute of Chemical Sciences and Engineering, École Polytechnique Fédérale de Lausanne (EPFL), Lausanne 1015, Switzerland; § Center for Materials Science and Nanotechnology (SMN), Department of Chemistry, 6305 University of Oslo, Oslo 0315, Norway; ∥ Hylleraas Centre for Quantum Molecular Sciences, Department of Chemistry, University of Oslo, Oslo N-0315, Norway; ⊥ Institute of Chemical and Bioengineering, Department of Chemistry and Applied Biosciences, 27219 ETH Zurich, Vladimir-Prelog-Weg 1, Zurich 8093, Switzerland; # Institute for Theoretical Chemistry, University of Stuttgart, Pfaffenwaldring 55, Stuttgart 70569, Germany; ¶ Department of Chemistry, University of Basel, Klingelbergstrasse 80, Basel 4056, Switzerland; ∇ Laboratory of Artificial Chemical Intelligence (LIAC), Institute of Chemical Sciences and Engineering, École Polytechnique Fédérale de Lausanne (EPFL), Lausanne 1015, Switzerland; ○ Department of Chemistry and Applied Biosciences, ETH Zurich, Vladimir-Prelog-Weg 2, Zurich 8093, Switzerland; ⧫ Max-Planck-Institut für Kohlenforschung, Kaiser-Wilhelm-Platz 1, Mülheim an der Ruhr 45470, Germany; †† Department of Chemistry, Quantum Chemistry, TU Darmstadt, Peter-Grünberg-Str. 4, Darmstadt 64287, Germany; 12 Laboratory of Computational Chemistry and Biochemistry, École Polytechnique Fédérale de Lausanne (EPFL), Lausanne 1015, Switzerland

## Abstract

Artificial intelligence (AI) and machine learning (ML) are rapidly reshaping the landscape of computational chemistry, offering new opportunities for accelerating catalyst discovery and deepening our understanding of chemical reactivity. This perspective highlights emerging methodologies ranging from machine learning potentials and reinforcement learning to generative AI and large language models that are poised to transform computational catalysis. We discuss challenges in developing robust molecular representations for transition-metal complexes, bridging mechanistic understanding with AI-driven predictions, and constructing reliable data sets that capture both successful and failed reactivity outcomes. By drawing on the authors’ practical experience across computational, experimental, and AI-driven domains, we emphasize the importance of integrating chemical intuition and methodological expertise with data-driven approaches while remaining open to serendipitous discoveries enabled by automation and self-driving laboratories. Ultimately, the future of computational catalysis lies in balancing human intuition with algorithmic power, leveraging AI not as a replacement but as an accelerator of chemical insight, mechanistic understanding, and catalyst design.

## Graphical abstract

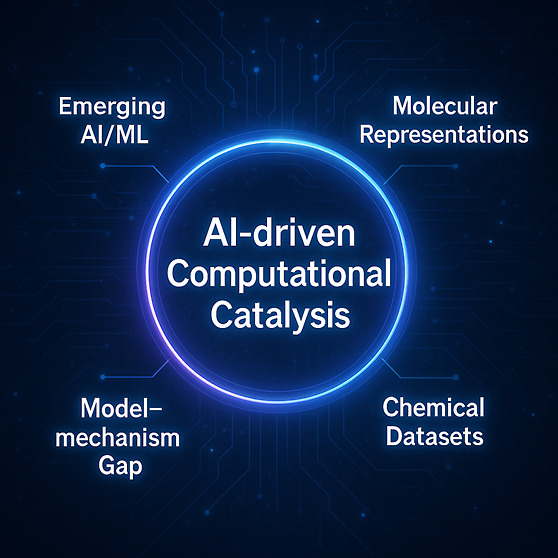

## 1 Introduction

Within less than a decade, machine learning (ML) and artificial intelligence (AI) have become pervasive in chemistry. A new branch of chemistry, often referred to as digital chemistry,^1^ has emerged at the intersection of experiment and computation. This field is dedicated to data science applications across chemistry in the broadest sense, extending well beyond the scope of traditional chemoinformatics. While pioneering work in this field is decades old,^2–4^ efforts during the 2010s facilitated by significant algorithmic and hardware advances, have prepared the ground for a data revolution in chemistry that we see now unfolding.^5–13^ What is different compared to previous (r)­evolutions is the rapid pace of development.^14^ This high innovation pressure appears to leave us hardly any time to step aside and evaluate the accomplishments. What have been the most important developments? How are they consolidated, up to the point where they need to be transformed into teaching material to educate the next generation of computational chemists with a focus on data science? What are the next steps to expect? What are the holy grails in the field? Thus, in this article we attempt to provide a perspective on the field and its context.

While AI-driven research on organic molecules and organic synthesis has entered in a more mature stage,^7,15,16^ —largely propelled by pharmaceutical applications^17–22^ —the fields of transition metal chemistry and heavy element chemistry remain comparatively underexplored.^23–26^ Technologies developed for molecules composed primarily of first- and second-row elements can play a pivotal role in advancing AI workflows for applications in homogeneous catalysis,^27,28^ spintronics,^29^ heavy element separations and capture.^30^ However, important challenges remain for transitioning such technologies to molecular complexes. Those have to do with emerging AI/ML methodologies such as generative AI and large language models (LLMs), molecular representations of metal–ligand structures, chemical insights into the AI workflows, and generation of reliable and diverse data sets.

In this perspective, we explore these emerging opportunities and challenges and discuss recent developments in AI/ML techniques and how they might transform molecular modeling, with a particular focus on molecular transition metal systems. We will have an emphasis on opportunities driven by actual practical experience of the authors, which is (in general) not documented in data sets and literature and therefore not accessible to AI-powered assistants. Hence, by contrast to a traditional review article covering recent literature on a specific topic, which is a perfect target for AI-powered robots (as long as they have access to all literature and without hallucinating about scientific facts), we understand this work as being part of an increasingly important push toward perspective and opinion pieces as they draw upon human intelligence and wisdom, not reachable by machines any time soon. Accordingly, we rely on a diverse background that ranges from traditional computational chemistry applications in catalysis and chemical reactivity to the development of chemical LLMs. While molecular catalysis is the primary focus of this perspective, we also highlight recent and particularly compelling examples of AI applications in heterogeneous catalysis whenever they provide meaningful extensions to our discussion. Figure 1 summarizes the topics that are discussed in the next sections.

**Figure 1:**
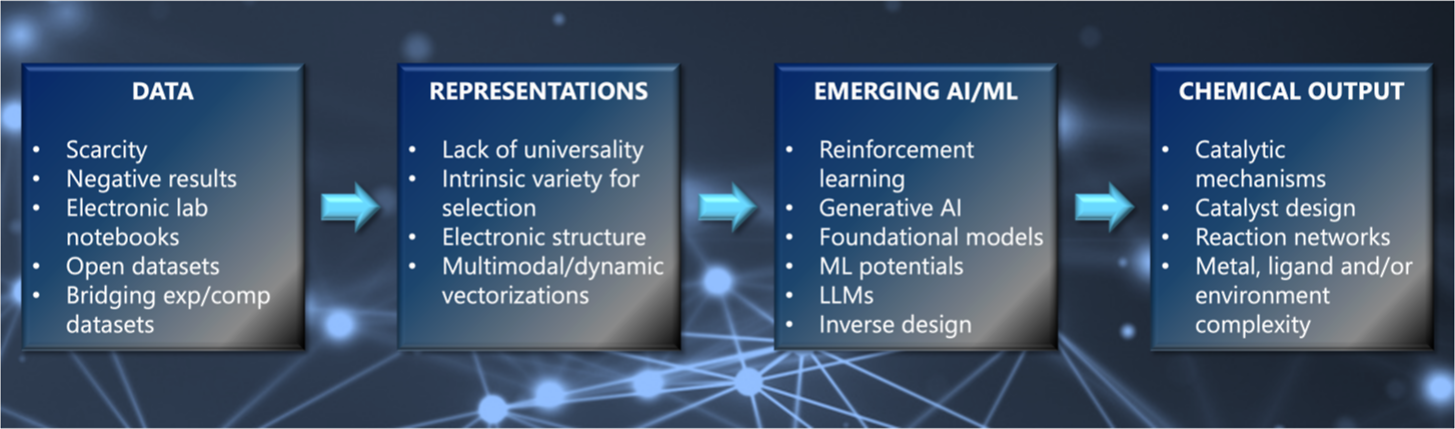
Schematic workflow of the four main topics explored in this perspective article.

## 2 Emerging AI/ML Methodologies

New methodologies such as transfer learning,^31–34^ reinforcement learning,^35–38^ self-supervised learning,^39^ generative AI,^40,41^ and foundation models^42–47^ are beginning to influence computational chemistry workflows. While there is still uncertainty about where these trends will lead, active learning, in particular, may soon replace the (manual or automated) generation of training sets—one of the most labor-intensive aspects of machine learning model development. For specific domains though, such as the ML-based computation of potential energy surfaces, there are already software suites which assist in this task, including PES-learn,^48^ Asparagus,^49^ ArcaNN,^50^ or DP-Gen^51^ to name a few. In this section, we discuss two emerging areas that are increasingly shaping computational chemistry workflows: machine learning interatomic potentials (MLIPs) and generative AI methods.

### 2.1 Machine Learning Interatomic Potentials vs Established Computational Methods

An increasingly prominent theme in molecular simulations is the emergence and growing influence of MLIPs and their prospect to replace traditional quantum chemical methods such as density functional theory (DFT).^52–57^ For many routine tasks such as conformer generation, reaction mechanism analysis, and energy profiling of drug-like molecules, MLIPs are beginning to match, and in some cases surpass DFT performance. Their advantage can be measured either in terms of accuracy, when trained on data from post-Hartree–Fock reference data (e.g., coupled-cluster), or in terms of speed, which can approach that of classical simulations. The rapid scaling of data sets and improvements in learning architectures suggest that many simulations currently handled with first-principles methods could soon be delegated to machine-learned models. In this view, DFT may increasingly serve as a data generation engine for training and validating machine learning models. This trend becomes even more evident with the emergence of general-purpose foundation models, such as MACE^58^ and UMA (the latter is trained on the OMOL2025 data set^59^). Unlike task-specific MLIPs, these models aim to capture broadly transferable representations of chemical interactions, enabling rapid adaptation to diverse molecular systems and tasks. Their promise lies in reducing the need for system-specific retraining while offering accuracy and scalability that approach, or in some cases exceed, traditional quantum methods.

This optimism can be balanced with an awareness of current limitations, which highlight opportunities for growth. Despite training on large data sets, MLIPs can exhibit “uncharted regions”^60^ in their energy landscapes, corresponding to portions of configuration space where the model produces unreliable or unphysical results.^61–64^ These issues are particularly pronounced in systems that fall outside the training distribution or that require fine control of electronic structure details. For instance, a computational campaign considered complete may need to be extended by months once it becomes clear that parts of the relevant potential energy surface are poorly represented. This points to a key limitation: while machine learning models always return a result, assessing the reliability of that result, particularly in underexplored regions of chemical space, remains a significant challenge. Recent progress in uncertainty estimation has the potential to elevate active learning for the exploration of complex potential energy surfaces, including transition metal complexes, with significant implications for catalyst design.^65–67^

We may draw a useful comparison to the early adoption of DFT itself. When DFT methods were first introduced, there was skepticism about their limitations and applicability to complex systems.^68^ Similar doubts are now being raised about MLIPs. While there will always be challenging cases such as multireference problems,^69,70^ or photochemistry,^71,72^ many everyday chemical simulations may eventually be handled by MLIPs with minimal loss in accuracy and significant gains in efficiency. Before reaching this level of maturity, computational chemists will have access to a rapidly growing array of semiempirical methods, including those based on tight-binding (TB) approximations to Kohn–Sham DFT.^73,74^ Much of the eventual success of DFT can be traced to the systematic identification of functional-specific failure modes, which provided confidence in their domain of applicability. Whether MLIPs will undergo a comparable process remains to be seen.

Although DFT has played a prominent role for various reasons, theoretical investigations of catalytic mechanisms, particularly in the case of transition metal catalysts, currently employ a range of higher-level electronic structure methods, either single-reference (e.g., coupled-cluster-based methods) or multireference. For systems with complicated electronic structures, multinuclear catalysts, complexes with noninnocent ligands, magnetic exchange interactions, reaction intermediates during bond formation and breaking possibly involving spin-state crossings, these methods may be necessary to reach a certain level of accuracy but they may also be desirable in themselves because they facilitate a level of insight that is not readily accessible by other means (e.g., analyzing the nature of multireference states). Here, a balanced view of the role of ML might be as a facilitator/accelerator of such approaches rather than as an alternative to them. Similarly, for heterogeneous catalysis, post-DFT electronic-structure methods for periodic systems (such as GW,^75^ periodic RPA,^76^ embedding approaches,^77^ and local-correlation methods,^78,79^ ) fulfill a comparable role by enhancing both the accuracy and interpretability of surface reaction mechanisms.

Protocols where ML is used in conjunction with classic quantum chemistry methods (e.g., in a Δ-ML fashion or through transfer learning),^31,80^ such as DFT or correlated methods, have shown promise to improve accuracy for challenging problems. Some examples include data-driven quantum chemical methodologies that utilize low-level quantum chemical data for the prediction of higher-level wave functions (e.g., from MP2 → CCSD­(T)^81–84^ or CASSCF → CASPT2 ^85^), which can be in principle extended to DLPNO–CCSD­(T) for larger systems, extrapolation of NEVPT2 dynamic correlation corrections to estimate higher level corrections, such as NEVPT2 → NEVPT4, or on the automated active space selection.^86,87^ These protocols can also be used in turn to train more computationally efficient models. Δ-ML and transfer learning combined with cluster-based parametrization strategies^88^ and/or molecular tailoring^89^ provide a roadmap to address problems in homogeneous and even heterogeneous catalysis.

Training of such models is typically limited to data sets of representative systems with few atoms, on which a large percentage of electron correlation is possible to be recovered, but it is questionable whether the results obtained using such data sets can be extrapolated to realistic systems. In contrast, parameters such as orbital entropy, spin populations, and related quantities can instead be used to represent the complexes in the training set. These parameters capture features that can be shared between both small and large systems, such as the local electronic structure around the metal center(s). In this way, the model can learn connections between correlation energies and these parameters, rather than relying directly on molecular geometries.

For computational chemists working with metalloenzymes, a central consideration is how ML can be incorporated into existing workflows of multilevel calculations (e.g., QM/MM) and facilitate improved embedding strategies for treatment of environmental effects and solvation spheres. We do not envisage–in the short term–a complete dismantling of established multicomponent workflows but anticipate ML approaches to provide unique solutions to sensitive or demanding steps. For example, a common bottleneck in QM/MM/MD calculations of metalloenzymes is the generation of appropriate force-field parameters for metallocofactors, which may even have to be changing from step to step when different catalytic intermediates are studied. Here ML/MLIPs could potentially be used to automate this step for metalloenzyme active sites, thus drastically accelerating the overall workflow of—otherwise conventional—multiscale simulations.^90–101^

### 2.2 Generative AI

Speculation about the future may also touch on more transformative ideas. One may imagine a future in which foundation models—trained on massive experimental and computational data sets—could bypass traditional simulation-based studies of molecular systems.^12,102^ In such a vision, we could imagine moving directly from a research question to an experimental observable, without explicitly modeling the molecular system. Though still aspirational, the emergence of self-driving laboratories,^103,104^ real-time experimental data streams, and closed-loop AI systems point in this direction.

AI-driven laboratories will also require synergy between experimental and digital chemists to elevate the strengths of machine learning and develop models easily accessible to nonexperts. Web services or easy-to-use applications will allow chemists to use such models without prior programming experience.

Diffusion and flow-matching models are powerful new generative tools in chemistry that directly operate on 3D molecular geometries, providing a natural interface with traditional quantum chemistry modeling.^105^ Unlike autoregressive models, they do not generate structures sequentially but instead evolve entire geometries in continuous space, which improves validity and facilitates modeling of interactions relevant to reactivity. While much of their early success has been in drug discovery,^106,107^ flow-matching models have been applied to generate transition-states,^108,109^ in inverse design of organocatalysts^110^ and enzymes,^111^ with recent extensions demonstrating their applicability to heterogeneous catalysis.^44^ These capabilities suggest that generative models based on diffusion or flow matching may open new avenues for interpretable catalyst design and mechanistic discovery.

As MLIPs continue to redefine how we model potential energy surfaces with quantum-level accuracy, a parallel frontier is emerging at a very different level of abstraction: foundation models. In contemporary computational chemistry, the term “foundation model” has come to encompass two complementary developments. The first includes broadly transferable MLIPs—discussed in the previous section—which serve as universal energy and force predictors across chemical and materials spaces. The second refers to large language models (LLMs) trained on extensive chemical, molecular, or materials corpora.^112–121^ Although both groups of models share underlying principles such as large-scale training, generalization, and cross-domain transferability, they differ fundamentally in their objectives, data modalities, and modes of interaction with chemical problems. While LLMs themselves still perform poorly in generating valid molecules or reactions from scratch, they are rapidly improving at interpreting, contextualizing, and reasoning about chemical information.^122^ These models are increasingly used by students and researchers for coding support, structure interpretation, and scientific ideation. LLMs have the potential to bridge traditional computational tools (such as DFT) with the broader research workflow, enabling more natural and agentic interactions with complex data pipelines.^123^ LLMs with specialized tools have shown promise for coding, processing of chemical information, and controlling lab automation.^112,115^

However, are foundation models a panacea? A cautionary analogy might be offered by the story of the Universal Force Field (UFF),^124^ which was once promoted as a general solution for all chemistry but ultimately fell short due to its lack of precision and transferability. The same risk applies to universal machine learning models: while the allure of generality is strong, chemistry continues to demand rigor, specificity, and domain expertise. Even as ML tools become more powerful, careful model validation and deep chemical intuition remain essential. But we should keep in mind that foundation models offer, in effect, an implicit universal projection of diverse electronic structures into a compact analytic representation, enabled through careful retraining and fine-tuning. In many ways, they can be viewed as a natural continuation of the vision behind UFF: the pursuit of a broadly applicable analytic model of fundamental physical interactions. What is different today is the nature of the representations themselves. Modern ML representations are implicit rather than explicit, which gives them a surprising capacity for extrapolation and allows them to be rapidly retrained into highly accurate, problem-specific models. In this sense, foundation models extend and refine the goal that UFF originally sought to achieve.

Equally important is the conceptual formulation of the scientific questions we ask. In heterogeneous catalysis, while developing an accurate potential energy surface remains essential, a central difficulty often also lies in selecting an appropriate system and representation to model from the outset. AI/ML tools can help accelerate discovery, but they do not replace the need for human insight in defining the scope and relevance of a problem. However, recent advances on multiagent architectures and literature search agents have shown promise in generating new, original knowledge and formulating self-improving research hypotheses for automating scientific discoveries.^125,126^

Clearly, a sense of both opportunity and caution must be exerted. AI and ML techniques are rapidly expanding the frontier of what is possible in computational chemistry, especially in areas like transition metal chemistry that have traditionally resisted automation. But the road ahead requires thoughtful integration of these tools with existing methodologies, careful attention to their limitations, and a commitment to preserving scientific rigor even in the face of accelerating automation.^127^ If pursued responsibly, these emerging technologies could dramatically enhance our ability to model, predict, and ultimately design the chemical systems of the future.

## 3 Molecular Representations

A complex and evolving challenge is how chemical systems are represented in a form suitable for computational modeling.^128,129^ Whether designing new machine learning models, interpreting experimental data, or predicting physical properties, the way we encode chemical information into molecular representations plays a central role in determining what questions can be asked and what answers can be trusted.

Choosing the “right” molecular representation remains an open problem, one deeply tied to the nature of the task at hand. In contrast to fields like computer vision or natural language processing, where inputs are relatively standardized (images, text), chemistry offers a puzzling range of possible representations: text-based formats like SMILES, graph-based molecular topologies,^130^ three-dimensional geometries, electron densities, and quantum mechanical wave functions, among others. One may use text-based representations to take advantage of LLMs as general-purpose feature extractors. With relatively few training examples, these models can adapt representations on the fly to new tasks or reactions.^131^ However, this flexibility comes at a cost, as these representations are often highly abstract, and their interpretability is limited.

The trade-offs between accuracy and interpretability, generality and specificity, are important to understand. One may advocate for dynamic or task-specific representations, learned in tandem with the data set as it is collected. However, such adaptive methods may obscure the underlying chemistry and make it more difficult to rationalize model behavior. Just as in human scientific communities, diversity of perspectives, described as the romantic, the pragmatic, and the artistic, can be a strength, allowing the field to pursue multiple lines of inquiry without prematurely standardizing on a single “best” approach.

The abundance of representations in chemistry is both a challenge and an opportunity. Unlike many areas of machine learning where data are prevectorized, chemistry permits—and requires—investigation into how the form of representation affects model outcomes, which makes machine learning in chemistry a particularly rich domain: it opens the door to investigating more complex problems than are typically addressed by off-the-shelf ML benchmarks.

Yet despite the promise of learned or abstract representations, hand-crafted, expert-defined features still play an important role.^132^ Especially in data-scarce regimes or for chemically challenging systems, such as transition metal complexes,^25,26,133–139^ or heterogeneous catalysts, domain knowledge remains crucial for designing meaningful inputs. As the field matures and access to experimental and computational data grows, we may see a convergence where learned representations become more powerful, efficient, and easy to use, while still respecting the chemical constraints and principles that underlie molecular behavior.

One should be cautious against the temptation to search for a single unifying representation. The needs of spectroscopy differ from those of reaction prediction; electron density offers different information than molecular graphs or SMILES strings. The representation should not just describe the data but it should also reflect the specific modeling goal. A good representation is one that enables extrapolation, generalization, and problem-specific reasoning. And depending on the task (predicting spin crossover,^140^ ,^141^ reactivity,^142^ or spectral lines^143^) different aspects of a molecule’s physical or electronic structure may become relevant.

From a computational chemistry standpoint, structure-based representations remain the default starting point. But even here, nuances arise. For instance, many descriptors assume a single, static conformation, whereas chemical reality often involves ensembles of rotamers, conformers, tautomeric forms or resonance structures.^144–146^ Thus, representations may need to move beyond idealized geometries to encode chemical states, incorporating information such as charge, spin multiplicity, solvation, or even temperature and concentration. Particularly in open-shell systems or complex electronic environments, these additional layers of description are critical for meaningful predictions.

In this context, electron density emerges as a particularly intriguing modality.^147–151^ Although it contains all the information needed to reconstruct molecular structure and properties, it is computationally expensive to calculate accurately and difficult to work with directly. In this sense, much of modern representation theory in chemistry can be seen as constructing reduced models that compress the full electronic description into something tractable and application-ready, such as, for example, the smooth overlap of atomic positions (SOAP) representation,^147^ which can be understood as a simplified easy-to-evaluate model of molecular electron densities.^150^

Looking ahead, the importance of multimodal representations is to be highlighted. In real chemical workflows, a molecule is rarely defined by a single modality. Structural data may be complemented by spectra, thermodynamic observables, or reaction yields. As such, the ability to combine different forms of information—text, graphs, images, numerical spectra—into a unified model offers a promising path forward. Multimodal machine learning frameworks, which can integrate and reason over diverse input types, may be key to unlocking more powerful and generalizable models. This approach also reflects experimental practice more accurately: a chemist rarely sees just a molecule but rather a chromatogram, an NMR spectrum, a color change in solution.

Ultimately, representation is not just about how data are encoded. It is also about how information is preserved, transformed, and used to answer meaningful scientific questions. While machine learning today leans heavily on large data sets to learn patterns, traditional quantum chemistry began from minimal assumptions and sought predictive power from first principles. Future methods will likely benefit from bridging these paradigms: integrating the rigor of physics-based models with the flexibility and scalability of data-driven approaches.^152,153^ This fusion could yield representations that are not only accurate and efficient, but also transparent and chemically meaningful, enabling both prediction and understanding in complex molecular systems.

## 4 Bridging the Model-Mechanism Gap

Returning to catalysis, the central theme of this perspective, we next consider one of its most enduring challenges: the elucidation of catalytic reaction mechanisms. From a computational perspective, mechanism discovery often demands years of effort, requiring not only the identification of the most probable pathway but also exploration of multiple competing steps.^154^ This naturally raises the question: how can we leverage machine learning, in particular physics-informed or knowledge-embedded approaches, to accelerate and enrich mechanistic studies? ^155,156^

Experimental practice in mechanistic investigations of homogeneous or enzymatic transition metal catalysts typically rests on correlating spectroscopic observables with structural parameters of isolatable intermediates. Beyond simple structural characterization, these spectroscopic observables exclusively report on electronic structure, which is crucial to rationalize differences in efficiencies or mechanistic pathways between different catalysts. Although a lot of emphasis has been placed on an energy-based view of computational investigations (potential energy surfaces, transition states), a more immediate and still invaluable goal would be the use of ML for proposing correlations between (experimental) spectroscopy such as EPR, XAS, and Mössbauer, and molecular/electronic structure. This can be a “final answer” in itself, but could also be seen as supplanting a major part of the work of computational chemists, the part involving hypothesizing/conceptualizing molecular models that would be subsequently evaluated for their spectroscopic properties via quantum chemical methods.

While AI continues to advance, we must not overlook the enduring importance of domain knowledge.^121,157,158^ Traditional organometallic catalysis, for instance, benefits from decades of mechanistic insights. Reaction mechanisms are typically described as a succession of established reaction steps, including oxidative addition, ligand dissociation, and transmetalation. Therefore, a way to approach the problem computationally is to determine the order of these steps and the catalyst structure, yielding the lowest energy pathway. Knowledge of these steps often allows domain experts to navigate complex systems more efficiently than those approaching the problem purely from theory or computation and serves as a valuable first filter, particularly when mapping out initial steps or identifying plausible intermediates. Techniques such as ML-assisted transition state searches^159^ and microkinetic modeling can then refine this initial scaffold, particularly when augmented by experimental kinetic data.^160^

For researchers less adept at mechanistic intuition, advances in reaction network modeling, such as those pioneered by groups working on automatic reaction exploration, can be transformative.^161^ Over time, these tools could evolve into powerful discovery frameworks, uncovering off-cycle pathways, decomposition channels, or unforeseen bifurcations in systems that resist traditional mechanistic analysis.

A productive path forward may lie less in further constraining models with additional physical or chemical principles and more in broadening access to high-quality, diverse data sets derived from both computation and experiment.^162,163^ For example, if one had infinite kinetic data sets,^164,165^ or well-characterized molecular simulations accounting for solvation and entropy,^166^ a data-driven approach could be sufficiently expressive without human-imposed biases.

However, this raises a provocative counterpoint: does encoding so much prior knowledge into our models limit the discovery potential of AI? Relying too heavily on textbook mechanisms, intuition, or handcrafted features risks reinforcing existing biases, driving models toward familiar solutions and potentially away from more innovative or unexpected ones. If large models are trained only on known chemistry, can they truly generalize or extrapolate?

The role of dynamics and entropy in computational studies of transition metal complexes is an underexplored but critical dimension. Traditional approaches (both computational and human-driven) often emphasize energy landscapes and static structures. Yet, bifurcating transition states, entropic barriers, and nonstatistical dynamics likely play a much larger role than typically appreciated.^167^ Capturing such behavior may require novel descriptors or models that treat mechanistic pathways as ensembles rather than linear sequences.

With increasing model complexity, interpretability re-emerges as a central consideration in ensuring scientific validity.^168,169^ Could a future model such as, for example, an LLM trained on domain-specific data, strike a balance between interpretability and prediction? Natural language interfaces to such models could actually improve transparency, allowing users to query mechanisms or compare alternatives in human-understandable terms. However, the application of LLMs or similar models in catalysis faces two important challenges. First, the data scarcity in this field prevents the development or fine-tuning of LLMs, while lower complexity statistical models such as decision tree ensembles or Gaussian processes are used more frequently.^170^ Second, the lack of clarity around the exact structure of the catalyst—specifically, how it can be characterized with limited information, typically restricted to reaction conditions, synthesis parameters, and averaged elemental descriptors from nominal loadings—makes it difficult to develop the kind of precise representation needed for concise embedding in an LLM. This is in contrast to fully defined molecular structures, which can be easily tokenized via SMILES.^171^

Ultimately, while AI will undoubtedly transform how we explore catalytic mechanisms, experimental validation will remain the final arbiter. Efforts in automation and self-driving laboratories,^103,104^ are a path toward fully integrated platforms for hypothesis generation, validation, and iterative improvement. Yet even here, some studies caution that smart sampling strategies (e.g., active learning) may be less impactful than how we represent the underlying chemical space.

We are best advised to remain thoughtful about the balance between automation and human input. While automation may accelerate exploration, the intuition honed through years of experimentation and theory still plays a pivotal role, especially in under-characterized or ill-defined systems, such as many homogeneous or heterogeneous catalysts. Moving forward, the field may benefit from embracing both extremes: large, expressive models trained on extensive data, and targeted, domain-informed approaches where prior knowledge provides critical context. Machine learning models could also be trained to capture chemists’ intuition in a similar way as has been attempted in the drug discovery space.^172,173^

In terms of catalyst optimization, emphasis is usually placed on the first coordination sphere of a transition metal ion. Bioinorganic catalysis however also encompasses metalloenzymes, and in this case the transferability of the developed approaches is not obvious. It is likely that quite distinct ML tools would have to be combined into multicomponent workflows, borrowing from more “locally focused” domains as well from domains focusing on directed enzyme evolution, recognizing that “textbook”-guided considerations of ligand optimization in standard coordination chemistry can quickly become even irrelevant when the “ligand” is a functionally important protein matrix. These approaches could be integrated in a bottom-up protocol for enzyme discovery acceleration, for example (re)­designing enzyme function.^174,175^ Distinct goals will have to be combined in order to construct a multidimensional surface correlating structural and electronic parameters of the active site with catalytic activity and with amino acid sequence, and ultimately to locate combinations that maximize overall function. Besides conventional conceptions of reactivity, repurposing natural or designing artificial metalloenzymes would need to consider multiple other factors, such as robustness, sensitivity of different parts of the system to specific conditions, optimization of substrate delivery pathways and proton channels, or even allostery for controlling selectivity and function, or for signaling between catalytic components (feedback).

Finally, in the realm of catalyst design, we anticipate significant breakthroughs in the coming years driven by inverse design strategies and generative models.^110,176,177^ The effectiveness of training generative models depends critically on the benchmark tasks they are designed for. While initial molecular design tasks focused on simplistic objectives such as molecular rediscovery and proxies such as quantitative estimates of drug likeness,^178^ benchmarks more tailored toward realistic targets have been developed.^179^ Other important aspects are the focus on sample-efficiency,^41^ and beating relevant baseline models such as genetic algorithms.

## 5 Chemical Datasets

Another difficulty in applying machine learning in catalysis research lies in data collection for the development of supervised learning models with predictive power. There is scarcity of computationally refined databases of transition metal complexes that machine learning can capitalize on,^59,180–182^ although there are ligand data sets that can be used for combinatorial expansions and genetic algorithms.^183–185^ Another known limitation is the lack of negative reaction outcomes that are not published by chemists, even though they are highly valuable to train machine learning models to avoid a positive reaction bias.^186,187^ A focus must therefore be put on encouraging and incentivizing chemists to also publish negative reaction outcomes, a culture shift which requires a long community effort.^188^ This can be facilitated with electronic lab notebooks, in which all data are stored in a systematic and concise format.^189^ Recent studies have shown that the explicit generation of negative reaction data can help the models to better distinguish between reactive and unreactive substrates.^190^ Such models can then be used to identify further negative reactions that can be executed with high-throughput experimentation equipment to augment current unbalanced data sets. As awareness of this topic grows within the field, we are confident that the community will continue to move in the right direction. Data from automation platforms will also likely contain a larger percentage of failed reactions. Another important aspect is how to format reaction data once they are recorded since the role of chemical species in reactions is often unclear. We envision that the usage of electronic lab notebooks and open reaction databases^191^ will provide more interoperable and reusable reaction data, facilitating machine learning efforts. Such database-oriented format might also be combined with user-friendly spreadsheet formats at the time of input.^192^ The development of more sophisticated models and data infrastructure enables the community to move beyond standard data sets, such as the United States Patent and Trademark Office (USPTO), which is dominated by industrially relevant reactions such as the Buchwald–Hartwig or Suzuki coupling. Making efficient use of these sparser data is therefore highly important, e.g. through transfer learning or the previously alluded to chemistry-informed models.^193^ Finally, the validation of reaction standardization platforms will critically depend on the involvement of experimental chemists. As the primary data providers, it is essential to maintain a careful balance by offering them a flexible recording format that does not constrain their workflow while ensuring the data remains useable for machine learning applications.

A critical challenge in modern chemical research lies in reconciling experimental and computational data sets, particularly in terms of their reliability, resolution, and mutual alignment, despite several “best practice” rules that have been recommended in the literature.^194–196^ For example, while data sets of computed activation energies^197–200^ have helped fuel model development, the end goal is to predict experimental values. For many reaction types, agreement between computation and experiment remains challenging due to many factors. Machine learning models that bridge the computed activation and experimental activation energies through delta or transfer learning is a promising strategy.^31,201^ Using MLIPs and free energy simulations^166^ can also enable the generation of higher quality data sets from computations.

## 6 Conclusions

A well-known old quote attributed to Eugene Wigner (but probably apocryphal) notes that “It is nice to know that the computer understands the problem, but I would like to understand the problem, too”.^202^ It is hard to believe that at Wigner’s time, a computer that delivered some numerical result could be considered to have understood this result. Certainly the quote was referring to the fact that numerical data alone, no matter how accurate and reliable, do not allow us to grasp a scientific problem. However, we have now approached a qualitatively new reality in computational science in general, where machine learning models do recognize patterns invisible to the human eye and maybe even incomprehensible by humans. Now, based on their “insights”, computers may actually take decision to perform new calculations or even experiments autonomously, and therefore, we have now really come to the situation where we can truly say “it is nice that the computer understands a problem, but how and to what degree can we understand it, too?”

In this perspective, we focused on how we can bridge such “insights” from chemical knowledge and intuition with the role of modern AI/ML in accelerating molecular discovery, particularly in catalysis. Machine learning has begun transforming how researchers navigate chemical space and identify candidates for new catalysts, yet significant challenges remain. Model performance often hinges on data quality, and while generative models, graph neural networks, and LLMs show promise, careful representation of chemical knowledge remains critical. Domain expertise may be leveraged to guide model training, but one must not restrict the ability of letting models discover novel patterns without human bias, pointing to trade-offs between interpretability, physical intuition, and empirical discovery, that will need to be balanced depending on the application and availability of data.

In bridging the gap between mechanistic understanding and AI-driven modeling, which is a foundational concern in catalysis, the rich body of mechanistic knowledge found in textbooks and chemical intuition could still serve as valuable inputs when designing machine learning workflows. The rather new promise of automated reaction network exploration with coupled microkinetic modeling, requires us to ponder the trade-offs between incorporating prior knowledge versus enabling models to learn from data alone. As the field moves toward self-driving laboratories and closed-loop discovery, a future can be envisioned where models can generate hypotheses, guide experiments, and build new knowledge autonomously. Yet, without interpretability, domain specificity, and experimental validation, scientific understanding may be compromised. The balance between automation and human insight remains a defining question for the future of AI in chemistry and catalysis. It is therefore essential to emphasize the role of scientific expertise, ensuring that AI remains a powerful tool to augment, rather than replace, human reasoning—contrary to the perception that it offers a “panacea” or an “effortless cure”.
